# Supplementary material for: WordCommentsAnalyzer: A windows software tool for qualitative research
Source: F1000Res. 2018 Sep 4;7:536. Originally published 2018 May 3. [Version 2] doi: 10.12688/f1000research.14819.2 (PMC6137411; doi:10.12688/f1000research.14819.2)
Supplement: Supplementary file 3 [file f1000research-7-17614-s0002.tgz › b2a79496-4ada-47c4-9682-a99ce867836d.zip!/Eight Iranian Health Journals/Journal of Research in Rehabilitation Sciences _5.docx]

© Journal of Research in Rehabilitation Sciences . This work is licensed under CC BY-NC 4.0 (https://creativecommons.org/licenses/by-nc/4.0/).

Volume & Issue: دوره 12، شماره 6: 1395

Title: مقایسه مهارت‏های اجتماعی کودکان 6 تا 9 ساله سخت شنوا با کاشت حلزون و سمعک در شهر شیراز

http://jrrs.mui.ac.ir/index.php/jrrs/article/view/2757

مقدمه: استفاده از وسایل کمک شنوایی مانند کاشت حلزون و سمعک، می‌تواند تأثیرات زیادی بر ابعاد مختلف زندگی افراد دارای آسیب شنوایی داشته باشد. هدف از انجام پژوهش حاضر، مقایسه مهارت‌های اجتماعی کودکان 6 تا 9 ساله سخت شنوا با سمعک و با کاشت حلزون بود. مواد و روش‌ها: در این مطالعه مقطعی- مقایسه‌ای، از پرسش‌نامه مهارت‌های اجتماعی Matson (Matson Evaluation of Social Skills with Youngsters یا MESSY) و نسخه سوم آزمون رشد زبان (Test of Language Development-3rd Edition یا 3-TOLD) برای ارزیابی مهارت‌های اجتماعی و رشد زبان 118 کودک دارای آسیب شنوایی (58 نفر با کاشت حلزون و 60 نفر با سمعک) استفاده شد. جهت تجزیه و تحلیل داده‌ها، آزمون تحلیل واریانس چند متغیره مورد استفاده قرار گرفت. یافته‌ها: با کنترل سطح رشد زبان، تفاوت معنی‌داری بین رشد اجتماعی کودکان با کاشت حلزون و کودکان دارای سمعک مشاهده شد (001/0 > P)؛ به طوری که کودکان کاشت حلزون در مقایسه با کودکان دارای سمعک، رفتارهای اجتماعی مناسب‌تر، پرخاشگری کمتر و مشکلات بیشتری را در روابط با همسالان نشان دادند (001/0 > P). نتیجه‎گیری: کودکان کاشت حلزون در مقایسه با کودکان دارای سمعک، مشکلات بیشتری را در رابطه با همسالان تجربه می‌کنند و مداخلات آموزشی و درمانی در این زمینه ضروری به نظر می‌رسد.

Title: مقایسه میزان بروز آسیب دیدگی بازیکنان فوتبالیست مرد جوان در چمن مصنوعی و طبیعی

http://jrrs.mui.ac.ir/index.php/jrrs/article/view/2814

مقدمه: به‌تازگی استفاده از چمن مصنوعی در بسیاری از مناطق جهان رایج شده است، اما در زمینه اثر چمن مصنوعی بر بروز آسیب‌های فوتبال، هنوز تردید وجود دارد و مطالعات نتایج متناقضی را گزارش نموده‌‌اند. بنابراین، هدف از انجام مطالعه حاضر، مقایسه بروز آسیب‌های بازیکنان جوان ایرانی در چمن طبیعی و مصنوعی بود. مواد و روش‌ها: 35 تیم رده سنی جوانان فوتبال ایران (980 بازیکن) در این تحقیق شرکت کردند. تعداد ساعات فعالیت تیم‌ها، نوع چمن زمین به همراه آسیب‌های بازیکنان در طول مدت مطالعه به ‌صورت آینده‌نگر توسط مربیان و کادر پزشکی تیم‌ها در طول یک فصل ثبت شد. آسیب‌هایی ثبت گردید که در تمرین یا مسابقه رخ‌ داده بود و بازیکن آسیب ‌دیده قادر به شرکت در جلسه تمرینی یا مسابقه روز بعد تیم نبود. از کادر پزشکی و مربیان تیم‌های هر دو گروه شاهد و مداخله درخواست گردید تا آسیب‌های بازیکنان را در فرم مربوط ثبت نمایند. یافته‌ها: طی 7 ماه مطالعه، در مجموع 26 هزار ساعت فعالیت و 118 آسیب ثبت گردید. نتایج آزمون Poisson نشان داد که بازیکنان روی زمین چمن طبیعی (04/4 آسیب در هر هزار ساعت)، به ‌طور معنی‌داری کمتر از چمن مصنوعی (64/7 آسیب در هر هزار ساعت) آسیب ‌دیدند (05/0 > P). همچنین، آسیب‌های زانو و مچ پا در چمن مصنوعی بیشتر از چمن طبیعی مشاهده شد (05/0 > P). میزان بروز آسیب‌های استفاده بیش از حد در چمن مصنوعی حدود دو برابر چمن طبیعی بود (05/0 > P). نتیجه‎گیری: با توجه به نتایج مطالعه حاضر، پیشنهاد می‌گردد مربیان و بازیکنان فوتبال جوان به بروز بیشتر آسیب‌ها در زمین‌های چمن مصنوعی توجه بیشتری نمایند و تدابیر لازم در جهت پیشگیری از آسیب‌ها در چمن مصنوعی اندیشیده شود.

Title: مقایسه تأثیر هشت هفته برنامه تمرینی مقاومتی، تعادلی و ترکیبی (مقاومتی- تعادلی) بر تعادل، راه رفتن و کیفیت زندگی بیماران مبتلا به مولتیپل اسکلروزیس: مداخله پیش‌آزمون- پس‌آزمون

http://jrrs.mui.ac.ir/index.php/jrrs/article/view/2798

مقدمه: تمرین درمانی، یکی از روش‌‌های مکمل درمانی برای توان‌بخشی بیماران مبتلا به مولتيپل اسكلروزيس (Multiple sclerosis یا MS) محسوب می‌شود. هدف از انجام مطالعه حاضر، مقایسه تأثیر هشت هفته تمرینات تعادلی، مقاومتی و ترکیبی (تعادلی- مقاومتی) بر تعادل، راه رفتن و کیفیت زندگی زنان مبتلا به MS بود. مواد و روش‌ها: تعداد 30 زن مبتلا به MS با میانگین سنی 1/4 ± 6/34 سال و با توجه به سطح ناتوانی، در سه گروه تمرینات تعادلی، مقاومتی و ترکیبی (تعادلی- مقاومتی) قرار گرفتند. آزمون تعادلی Berg (Berg Balance Scale یا BBS)، آزمون مسافت 6 دقیقه راه رفتن، پرسش‌نامه کيفيت زندگي بیماران مبتلا به MS (54-Multiple Sclerosis Quality of Life یا 54-MSQOL) و شاخص وضعیت گسترش ناتوانی (Expanded Disability Status Scale یا EDSS) به ترتیب برای سنجش تعادل، راه رفتن، کیفیت زندگی و میزان ناتوانی شرکت کنندگان استفاده گردید. بیماران به مدت هشت هفته تمرینات مورد نظر را انجام دادند. آزمون‌های ANOVA، Tukey و Paired t جهت تجزیه و تحلیل داده‌ها مورد استفاده قرار گرفت. یافته‌ها: تمرینات تعادلی و مقاومتی منجر به بهبود نمرات تعادل شد و همچنین تمرینات مقاومتی و ترکیبی باعث بهبود راه رفتن گردید.  اما بر کیفیت زندگی بيماران تأثير قابل ملاحظه اي نداشت.نتیجه‎گیری: به دلیل اثربخشی بیشتر تمرینات تعادلی و مقاومتی بر تعادل و تمرینات مقاومتی و ترکیبی بر راه رفتن استفاده از این تمرینات به عنوان روش مکمل توان‌بخشی، در بیماران مبتلا به MS ارجحیت دارد

Title: بررسی اثربخشی پروتکل جامع تمرینی عضلات ستون فقرات با و بدون تحریک الکتریکی عملکردی در افراد سالمند دارای ناهنجاری ‌هایپرکایفوزیس عملکردی

http://jrrs.mui.ac.ir/index.php/jrrs/article/view/2794

مقدمه: هدف از انجام پژوهش حاضر، بررسی اثربخشی پروتکل جامع تمرینی عضلات ستون فقرات با و بدون تحریک الکتریکی عملکردی در افراد سالمند دارای ناهنجاری هایپرکایفوزیس عملکردی بود. مواد و روش‌ها: در این مطالعه نیمه تجربی، 45 نفر از زنان دارای هایپرکایفوز عملکردی بودند که به صورت تصادفی در سه گروه 15 نفره شاهد، گروه تمرینات جامع تمرینی با تحریک الکتریکی عملکردی و گروه تمرینات جامع تمرینی بدون تحریک الکتریکی عملکردی قرار گرفتند. قبل و بعد از اتمام تمرینات، از همه آزمودنی‌ها جهت بررسی میزان زاویه کایفوز (با استفاده از روش رادیوگرافی Cobb) آزمون به عمل آمد. سپس، آزمودنی‌ها به مدت 8 هفته و هفته‌ای 3 جلسه (هر جلسه به مدت 45 دقیقه) تمرینات جامع تمرینی با و بدون تحریک الکتریکی عملکردی انجام دادند. جهت تجزیه و تحلیل داده‌ها ابتدا به منظور بررسی درون گروهی از آزمون t وابسته و از آزمون کوواریانس به منظور بررسی تفاوت بین گروهی استفاده شد. یافته‌ها: پس از یک دوره تمرینات جامع تمرینی با و بدون تحریک الکتریکی عملکردی، نسبت به گروه شاهد تفاوت معنی‌داری در میزان زاویه کایفوز وجود داشت (050/0 > P). همچنین، در بررسی تفاوت بین گروهی نیز نتایج نشان دهنده عدم تفاوت معنی‌دار بین گروه تمرینات جامع تمرینی با تحریک الکتریکی عملکردی و بدون تحریک الکتریکی عملکردی بود (050/0 ≤ P). نتیجه‎گیری: با توجه به یافته‌های تحقیق، پیشنهاد می‌شود که تمرینات جامع تمرینی جهت بهبود میزان زاویه کایفوز و پیشگیری از عوارض آن در این قشر از جامعه، در کنار سایر برنامه‌های درمانی گنجانده شود.

Title: تأثیر هشت هفته تمرینات صندلی بر عرض و طول گام بزرگسالان مبتلابه فلج مغزی پاراپلژی

http://jrrs.mui.ac.ir/index.php/jrrs/article/view/2818

مقدمه: عوامل منفی بسیاری در بیماران مبتلا به فلج مغزى وجود دارد که موجب انحراف مبتلایان از الگوی طببعی راه رفتن می‌شود. توانایی راه رفتن، یکی از شاخص‌های تعیین استقلال در فعالیت‌های روزمره این بیماران به شمار می‌رود. از این‌رو، بررسی ویژگی‌های کینماتیک راه رفتن (طول گام، عرض گام، سرعت و تواتر گام‌برداری) مورد توجه محققان قرار گرفته است. هدف از انجام تحقیق حاضر، بررسي تأثیر تمرينات صندلی بر طول و عرض گام در افراد مبتلا به فلج مغزی بود. مواد و روش‌ها: 40 زن و مرد مبتلابه فلج مغزی پاراپلژی به صورت هدفمند و با تعداد مساوی در دو گروه شاهد و تجربی قرار گرفتند. قبل و بعد از یک دوره تمرینات صندلی هشت هفته‌ای، شاخص‌های کینماتیکی طول و عرض گام با سیستم سه بعدی آنالیز حرکت (Motion Analysis) اندازه‌گیری شد. از آزمون Repeated measures ANOVA جهت تجزیه و تحلیل داده‌ها در سطح معنی‌داری 05/0 > P استفاده گردید. یافته‌ها: طول و عرض گام در گروه با تمرینات صندلی بهبود یافت (001/0 = P) و در بررسی‌های بین گروهی تفاوت معنی‌داری بین دو گروه در طول (020/0 = P) و عرض گام (042/0 = P) مشاهده گردید. نتیجه‎گیری: به نظر می‌رسد که تمرینات صندلی منجر به بهبود طول و عرض گام در افراد مبتلا به پاراپلژی می‌شود. بنابراین، انجام چنین تمریناتی در جهت بهبود مهارت گام‌برداری این بیماران توصیه می‌شود.

Title: ارتباط چشم ساکن و عملکرد حرکتی در کودکان اختلال هماهنگی رشدی

http://jrrs.mui.ac.ir/index.php/jrrs/article/view/2773

مقدمه: آخرین تثبیت بینایی قبل از آغاز حرکت را چشم ساکن گویند. تحقیقات نشان داده‌ است که چشم ساکن با سطح بالایی از خبرگی و عملکرد مرتبط است. بنابراین، هدف از انجام مطالعه حاضر، بررسی ارتباط چشم ساکن و عملکرد حرکتی کودکان مبتلا به اختلال هماهنگی رشدی بود. مواد و روش‌ها: در این مطالعه توصیفی- همبستگی، 30 کودک 7 تا 14 ساله شهر اصفهان به صورت نمونه‌گیری خوشه‌ای چند مرحله‌ای انتخاب شدند. شرکت کنندگان در مطالعه حاضر تکلیف پرتاب کردن و دریافت کردن را 10 مرتبه انجام دادند. هنگام اجرای تکلیف مورد نظر، داده‌های چشم ساکن توسط دستگاه ردیابی چشم، ثبت گردید. به منظور تعیین نرمال بودن داده‌ها، از آزمون Shapiro-Wilk و برای تعیین ارتباط بین متغیرهای تحقیق از ضریب همبستگی Pearson استفاده شد. یافته‌ها: بین عملکرد گرفتن با آغاز چشم ساکن ارتباط منفی معنی‌دار (001/0> P، 838/0- = r)، بین عملکرد گرفتن با پایان چشم ساکن (044/0 = P، 370/0 = r) و دوره چشم ساکن (0001/0> P، 849/0 = r) ارتباط مثبت معنی‌داری وجود داشت. نتیجه‎گیری: به طور کلی، متغیر چشم ساکن یکی از متغیرهای مؤثر و مرتبط با عملکرد حرکتی در کودکان دارای اختلال هماهنگی رشدی است.

Title: مقایسه یادگیری ضمنی توالی حرکتی در کودکان با و بدون اختلال هماهنگی رشدی (DCD)

http://jrrs.mui.ac.ir/index.php/jrrs/article/view/2792

مقدمه: تحقیق حاضر با هدف بررسی یادگیری توالی حرکتی ضمنی در کودکان با و بدون اختلال رشدی انجام شد. مواد و روش‌ها: جهت مقایسه یادگیری حرکتی- ضمنی، نرم‌افزار کامپیوتری تحت عنوان زمان واکنش متوالی (SRT یا Serial reaction time) در محیط برنامه‌نویسی C++ طراحی و مورد استفاده قرار گرفت. در تحقیق حاضر 24 کودک مشارکت داشتند که به دو گروه با (12 نفر) و بدون اختلال هماهنگی رشدی (12 نفر) تقسیم شدند و طی دو روز به فاصله 24 ساعت، 10 بلوک حرکتی را تمرین کردند. در ابتدای آزمون به یاد گیرنده‌ها در مورد ترتیب ظهور محرک‌ها هیچ گونه توضیحی داده نشد و یادگیری حرکتی آن‌ها به صورت ضمنی اتفاق می‌افتاد. داده‌ها بااستفاده از آزمون‌های آماری t مستقل و ANOVA تجزیه و تحلیل شد. یافته‌ها: گروه کودکان دارای اختلال هماهنگی رشدی، در مؤلفه سرعت و دقت یادگیری توالی حرکتی ضمنی تفاوت معنی‌داری با گروه همتایان سالم نداشتند (235/0 = P، 072/0 = P). با این حال، اثرات بین آزمودنی‌ها در طول بلوک‌های تکلیف توالی حرکتی مرحله اکتساب نشان دهنده تفاوت بین کودکان با و بدون اختلال رشدی در هر دو متغیر سرعت و دقت بود (001/0 > P). نتیجه‎گیری: به نظر می‌رسد که اگرچه کودکان دارای اختلال هماهنگی رشدی مشکلاتی در اکتساب و یادگیری مهارت‌های حرکتی روزمره دارند، اما توالی حرکتی ضمنی در آن‌ها بی‌نقص است. از این‌رو، در روند آموزش و توانبخشی کودکان دارای اختلال هماهنگی رشدی می‌توان از مزایای یادگیری ضمنی بهره برد.

Title: مروری نظام‌مند بر مقایسه کاشت حلزون‌های تک کاناله با چند کاناله از نظر استراتژی کدگذاری و درک گفتار

http://jrrs.mui.ac.ir/index.php/jrrs/article/view/2799

مقدمه: در سیستم‌های کاشت حلزون، واژه کانال به تعداد محل‌های تحریکی در گوش داخلی و یا حلزون دلالت داشته و به این شکل دامنه فرکانسی و یا زیر و بمی تعیین می‌گردد. سیستم‌های کاشت حلزون چند کاناله برخلاف کاشت حلزون تک کاناله، سیگنال ورودی را به باندهای فرکانسی متفاوتی تقسیم کرده و به مکان‌های مختلف تحریکی معین در گوش داخلی انتقال می‌دهد. داشتن محل‌های تحریکی بیشتر، 2 هدف اصلی را به دنبال دارد: 1- از آن‌جایی که فیبرهای عصب شنوایی در حلزون به صورت تونوتوپیک سازمان‌دهی شده است، تعداد الکترود بیشتر تفکیک فرکانسی بهتری را ارایه می‌کند. 2- اگر در حلزون مناطقی وجود داشته باشد که به تحریک الکتریکی به طور نامناسب و یا به هیچ وقت پاسخ ندهد، در برنامه‌ریزی دستگاه از تحریک آن مکان‌ها اجتناب می‌شود و اجزای فرکانسی ورودی پردازش شده، با تحریک همراه می‌شود. هدف از انجام مطالعه حاضر، مقایسه کاشت حلزون تک کاناله با چند کاناله از نظر استراتژی کدگذاری فرکانسی و اثر آن بر درک گفتار افراد دریافت کننده سیستم‌های کاشت حلزون بود. مواد و روش‌ها: مطالعه حاضر به وسیله بررسی پایگاه‌های علمی (Pubmed، Science direct، Google scholar) در بازه زمانی 2016-1965 با استفاده از واژگان مرتبط با موضوع انجام شد و مقالات با توجه به معیارهای ورود و خروج انتخاب گردید. یافته‌ها: کاشت حلزون‌های تک کاناله فرکانس را بر اساس سرعت شلیک ایمپالس‌های الکتریکی کدبندی می‌کند. کاشت حلزون‌های چند کاناله از نظریه استراتژی مکانی برای کدبندی فرکانسی استفاده می‌کند که در آن فرکانس‌های مختلف سیگنال شنوایی جداسازی شده و به شکل تونوتوپیک در طول در ازای حلزون، از طریق آرایه الکترودی ارایه می‌شود. کدگذاری مکانی و زمانی فرکانس‌های صدا می‌تواند تا حدودی به وسیله تحریک چند کاناله عصب شنوایی، حفظ و تکرار شود. در کاشت حلزون چند کاناله استراتژی کدگذاری دارای 2 مدل استخراج ویژگی و شکل موج می‌باشد. همچنین، درک گفتار حاصل از سیستم‌های تک کاناله و چند کاناله بررسی شد. نتیجه‎گیری: با توجه به یافته‌ها، می‌توان نتیجه گرفت که سیستم‌های کاشت حلزون تک کاناله با کدگذاری زمانی فرکانسی، نمی‌تواند به میزان کافی اطلاعات گفتاری را منتقل کند؛ در حالی که کاشت حلزون‌های چند کاناله به اندازه کافی شباهت به نقشه تونوتوپیک حلزون داشته و فهم گفتار در آن نسبت به وسایل تک کاناله بیشتر است.
